# Remaining Useful Life Prediction for Bearings Across Domains via a Subdomain Adaptation Network Driven by Spectral Clustering

**DOI:** 10.3390/s25226919

**Published:** 2025-11-12

**Authors:** Zhiqing Xu, Christopher W. K. Chow, Md. Mizanur Rahman, Raufdeen Rameezdeen, Yee Wei Law

**Affiliations:** Sustainable Infrastructure and Resource Management (SIRM), UniSA STEM, University of South Australia, Mawson Lakes, Adelaide, SA 5095, Australia

**Keywords:** remaining useful life prediction, spectral clustering, subdomain alignment, subdomain adaptation

## Abstract

Accurate *remaining useful life* (RUL) prediction of bearings is essential, as bearing failures compromise operational safety. However, distribution discrepancies caused by varying working conditions often degrade prediction performance. *Domain adaptation* (DA) has been widely used to mitigate this issue, but most DA methods align feature distributions on a global scale, overlooking fine-grained discrepancies within the same domain. *Subdomain adaptation* (SDA) offers a promising alternative by aligning feature distributions at a subdomain level. Despite its potential, existing SDA methods often use fixed subdomain boundaries, overlook the unequal importance of subdomains, and lack clustering mechanisms for similar features. These limitations hinder further improvements in RUL prediction accuracy. To address these issues, this paper proposes a novel model, *subdomain adaptation network driven by spectral clustering* (SC-SAN), which dynamically adjusts subdomain boundaries, assigns higher weights to key features, and clusters similar features during model training. The effectiveness of SC-SAN is validated through ablation, comparison and generalization experiments on the XJTU-SY and PRONOSTIA datasets. Experimental results show that SC-SAN achieves an average MAE of 0.1009 and RMSE of 0.1231 across two datasets, representing reductions of 19.86% and 23.41%, respectively, compared to existing state-of-the-art methods.

## 1. Introduction

Bearings are critical components of modern industrial equipment, and their failures can lead to severe economic losses and potential safety hazards [1,2]. Therefore, predicting the RUL of bearings is crucial to achieving predictive maintenance [3]. In recent years, data-driven methods have achieved success not only in bearing RUL prediction but also in other mechanical systems due to their ability to extract features directly from raw data [4,5]. For example, Spirto et al. [6] employed neural networks for gear fault detection. Similarly, Babak et al. [7,8] proposed a stochastic model for the diagnosis of electrical equipment. However, in real-world scenarios, accurate RUL prediction remains challenging because the feature distributions of training and testing data may differ due to variations in machine manufacturing and working conditions [9,10].

To minimize the feature distribution differences in bearing RUL prediction, DA has emerged as an effective method [11]. DA aims to align feature distributions between training and testing data, enabling models trained on source domain data to generalize effectively to target domain data without requiring labeled data from the target domain. Popular DA methods, such as adversarial-based methods [12] and metric-based methods [13], have successfully improved RUL prediction accuracy across different domains. However, these methods often assume that the entire source and target domains experience the same type of domain shift, resulting in global-scale alignment.

Global-scale alignment overlooks fine-grained discrepancies in feature distributions, which are crucial for accurate RUL predictions [14]. In bearing RUL prediction, features evolve across distinct health stages, and global alignment may incorrectly match features from different stages, leading to negative transfer. In contrast, local-scale alignment addresses this problem by grouping features with similar health conditions and aligning them separately, thereby preserving stage-specific patterns and temporal progression. For example, in temperature prediction tasks, short-term fluctuations may reflect hourly weather variations, whereas long-term trends capture seasonal changes [15]. Globally aligning all data would mix short- and long-term patterns, reducing prediction accuracy. Similarly, in bearing RUL prediction, aligning features from early, mid, and late degradation stages separately allows the model to capture stage-specific degradation patterns, improving transferability and prediction performance.

To achieve local-scale alignment, SDA has been proposed as a potential solution. Although SDA methods improve RUL prediction accuracy compared to traditional DA methods through subdomain-level alignment, three key limitations still hinder their potential to further enhance prediction performance.

**Figure 1:**
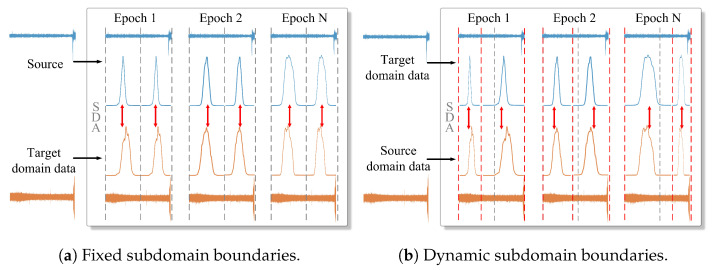
Visualization of subdomain boundaries. (**a**) The blue and orange curves depict the distributions of the source and target domains, with red arrows illustrating the process of reducing discrepancies between them. (**b**) Black dashed lines indicate the initial subdomain boundaries, while red dashed lines show the boundaries dynamically adjusted during each epoch.

(1) **Static subdomain division:** many SDA methods divide subdomains prior to the alignment process, resulting in fixed subdomain boundaries, as shown in Figure 1a. However, feature distributions often evolve during training, making these predefined fixed subdomain boundaries inaccurate [16]. For example, some features may shift across subdomains during training but remain incorrectly assigned to their original subdomains, as shown in Figure 1b. Without real-time updates to adjust these boundaries, models struggle to adapt to evolving feature spaces, hindering fine-grained subdomain alignment.

(2) **Unequal importance of subdomains:** different subdomains contribute unequally to SDA. For example, some subdomains represent the healthy stage of the bearing, while others represent the degradation stage [17]. During domain alignment, subdomains located in the degradation stage offer more degradation-related patterns than those in the healthy stage [18]. Therefore, it is crucial to assign greater importance to subdomains that represent the degradation stage during SDA. Treating all subdomains equally may lead to ineffective knowledge transfer and even negative transfer effects [19].

(3) **Fuzzy subdomain boundaries:** most SDA methods fail to encourage the clustering of features with similar distributions while separating features with dissimilar distributions during training. This often causes subdomains to be divided based on disordered feature distributions. Introducing a clustering mechanism to group similar features ensures that subdomains are divided based on well-clustered feature distributions, enabling precise subdomain division.

To address these three limitations, this paper proposes a novel RUL prediction model called SC-SAN, designed as an end-to-end model based on SDA. SC-SAN consists of a backbone network and three auxiliary modules. The backbone network aims to encode features and map them to prediction outputs. The three auxiliary modules are: ① a *temporal weight* (TW) generator, which assigns different weights to features using a normalized time-scalar function; ② a *spectral clustering* (SC) module, which groups similar features and separates dissimilar features during training; and ③ an SDA module, which performs subdomain-level alignment.

The main contributions of this paper can be summarized as follows:

1. The proposed SC-SAN dynamically adjusts subdomain boundaries during the training process, achieving fine-grained subdomain alignment.
2. SC-SAN generates a normalized time-scalar function to assign greater importance to degradation-related features during SDA, facilitating accurate RUL prediction.
3. SC-SAN incorporates a clustering mechanism into the parameter update process, guiding the model to update parameters in a way that groups similar features while separating dissimilar features, ensuring precise subdomain division.

The paper is organized as follows. Section 2 reviews related work on DA and SDA for RUL prediction. Section 3 explains the implementation of the proposed SC-SAN. Section 4 describes a case study on RUL prediction using SC-SAN and evaluates it with standard metrics. Section 6 concludes the paper.

## 2. Related Work

For bearing RUL prediction problems, extensive research has been conducted on DA. This section first briefly reviews the DA and SDA methods in RUL prediction, followed by a comparison with related works to highlight the contributions of the proposed method. Some methods mentioned in this section are listed in Table 1 and compared in Section 4, with experimental results presented in Section 4.6.

### 2.1. Domain Adaptation for RUL Prediction

In industrial RUL prediction, labeled data is often scarce [29]. DA addresses this by aligning feature distributions between source and target domains, enabling models trained on source data to generalize to target data. DA methods are typically categorized as ① metric-based methods, and ② adversarial-based methods [26].

**Metric-based methods** leverage metrics such as *maximum mean discrepancy* (MMD), *multi-kernel maximum mean discrepancy* (MK-MMD) [30], and *Wasserstein distance* (WD) [31] to reduce discrepancies between source and target feature distributions. For example, Cheng et al. [20] proposed *transferable convolutional neural network* (TCNN), an end-to-end model using MK-MMD, while Dong et al. [21] introduced *transferable LSTM multi-channel attention network* (TLMAN), a two-stage model based on MMD. Although effective in capturing global domain-invariant features, metric-based methods may neglect stage-specific differences, limiting the transfer of detailed domain knowledge.

**Adversarial-based methods** learn domain-invariant features through adversarial training [32,33]. Unlike metric-based methods, adversarial methods directly modify feature representations to achieve domain alignment [34]. Since Costa et al. [35] developed a *domain adversarial neural network* (DANN) and demonstrated its effectiveness in RUL prediction, DANN has become the baseline model for adversarial-based methods. Building on DANN, Zhuang et al. [22] proposed *metric adversarial domain adaptation* (MADA), which integrates a positive pair matching module from contrastive learning with DANN, significantly improving RUL prediction accuracy. Similarly, Dong et al. [23] introduced *multi-constrained domain adaptation* (MCDA), which combines a state discrimination module with DANN, enhancing RUL prediction performance. Despite their advantages, adversarial-based methods often require complex architectures and careful hyperparameter tuning, posing significant challenges for practical applications.

### 2.2. Subdomain Adaptation for RUL Prediction

SDA aims to align subdomains between the source and target domains at local scales. In fault diagnosis (classification) tasks, subdomains are typically predefined based on discrete labels (fault types). However, in RUL prediction (regression) tasks, the continuous nature of labels poses significant challenges for effective subdomain division. According to different strategies for subdomain division, the implementation of SDA in RUL prediction tasks can be categorized into two types: ① SDA based on two-stage models; ② SDA based on end-to-end models.

**SDA based on two-stage models** typically divide raw data into health stages using clustering algorithms [24], similarity metrics [25], or neural networks [26], and then aligns source and target features within each stage. For example, *Graph-embedded subdomain adaptation network* (GSAN) [24] leverages local manifold distributions to define subdomains and matches similar source–target pairs, *hierarchical adaptive multistage degradation network* (HAMDN) [25] segments data into three stages and aligns them using MMD, and *deep subdomain adaptation network with weighted multi-source domain* (DSAN-WM) [26] employs autoencoders to assign target samples to subdomains for alignment. While these methods are straightforward, their reliance on fixed subdomain boundaries can lead to errors when feature distributions evolve over time.

**SDA based on end-to-end models** involve constructing discrete labels. The feature distributions of the source and target subdomains are dynamically adjusted by minimizing the gap between the pseudo-labels output from the target domain and the constructed discrete labels of the source domain. For example, *deep subdomain adaptive regression network* (DSARN) [27] divides the source domain into 10 subdomains using the floor function to discretize continuous labels, then generates target pseudo-labels via softmax and floor operations for alignment. Similarly, *local weighted deep sub-domain adaptation network* (LWSAN) [28] applies the same pseudo-label strategy to adaptively align subdomains. Although these end-to-end models effectively achieve local-scale domain alignment by adaptively adjusting subdomain boundaries for target domain, the approach of dividing the source domain through time domain discretization relies on overly simplified assumptions and fails to account for the interactions between different subdomains [36].

### 2.3. Comparison with Related Works

The following sections compare the SC-SAN with related works to highlight the contributions of the proposed method.

**Comparison with DA models**: MADA and MCDA improved DANN by adding a positive pair module and a state discrimination module, respectively. These improvements are reflected in the loss function by adding regularization terms, significantly improving RUL prediction accuracy. Inspired by these models, we added an SDA module in the backbone network that serves as a regularization term in the loss function to enable fine-grained domain alignment.

**Comparison with SDA models**: Two-stage SDA models [24,25,26] rely on fixed subdomain boundaries, which causes errors introduced during the subdomain division phase to inevitably propagate and accumulate during the subdomain alignment phase. In contrast, end-to-end SDA models [27,28] based on pseudo-label learning can adaptively perform subdomain alignment, but their reliance on the discretization assumption limits their generalization capability. To address these problems, we integrate a clustering mechanism into the model training process, enabling adaptive identification of subdomain boundaries without requiring assumptions. Additionally, the proposed model introduces a TW generator that embeds a normalized temporal scalar function into the SDA framework. This module dynamically assigns weights to features according to their degradation stage, thereby highlighting those that are more relevant to the degradation process.

**Comparison with advanced models**: Besides the aforementioned methods that assume available source domains and sufficient samples, recent studies have explored challenging scenarios such as small-sample, unlabeled data and source-free domain adaptation. For example, Zhang et al. [37] proposed an adaptive RUL prediction method for single batteries under unlabeled small-sample data and parameter uncertainty, while Li et al. [38] investigated source-free domain adaptation and demonstrated promising results.

In addition, recent models have been developed to handle varying working conditions and cross-domain RUL prediction [39,40]. However, most of these methods still face limitations regarding feature distribution differences, model complexity, and training efficiency. Specifically, existing DA models mainly emphasize global alignment or rely on unstable adversarial learning, whereas current SDA models often use rigid subdomain boundaries. In contrast, the proposed SC-SAN achieves joint global–local alignment through adaptive subdomain discovery guided by temporal degradation patterns. Furthermore, unlike adversarial-based and two-stage SDA models that introduce extra discriminators or sequential optimization, SC-SAN adopts a lightweight unified framework that jointly optimizes clustering, temporal weighting, and alignment. Finally, SC-SAN performs single-stage online clustering-assisted alignment, enabling faster and more stable convergence with lower computational cost.

## 3. The Proposed Method

**Figure 2:**
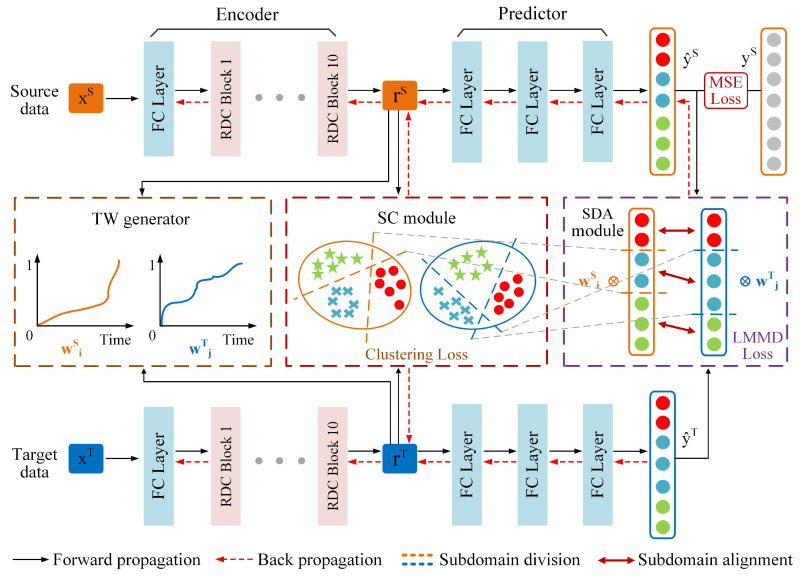
The architecture of the proposed SC-SAN, where stars, dots, and cross signs indicate different subdomains.

As illustrated in Figure 2, the proposed SC-SAN consists of a backbone network and three auxiliary modules: ① a backbone RUL prediction network; ② a TW generator; ③ an SC module; and ④ an SDA module.

Compared with existing SDA methods that rely on fixed subdomain boundaries, overlook the unequal importance of subdomains, and lack structural clustering constraints for feature grouping, SC-SAN introduces three auxiliary modules to overcome these limitations. Specifically, the TW generator constructs a normalized time indicator to adaptively assign different weights to different degradation stages. The SC module enforces clustering-aware constraints to group similar features and separate dissimilar ones, enabling dynamic adjustment of subdomain boundaries based on the evolving feature distribution. The SDA module aligns the feature distributions among the dynamically generated subdomains, facilitating robust cross-domain knowledge transfer. This section details the functions and implementations of each component. For clarity, all frequently used symbols in this section are summarized in Table 2.

### 3.1. The Backbone RUL Prediction Network

The proposed backbone RUL prediction network comprises two components: an Encoder and a Predictor. The Encoder reduces the feature dimensions of raw data to facilitate downstream tasks such as RUL prediction and SDA. Specifically, it transforms the input data $x\in R^{N\times M}$ into feature representations $r\in R^{N\times V}$, where V<M. The definitions of *N*, *M*, and *V* are detailed in Table 2. The Predictor then maps these learned representations *r* to RUL prediction values $\tilde{y}\in R^{N\times 1}$, where each value in $\tilde{y}$ corresponds to a predicted RUL at each time step. The structure and function of each component are detailed in the following.

**Figure 3:**
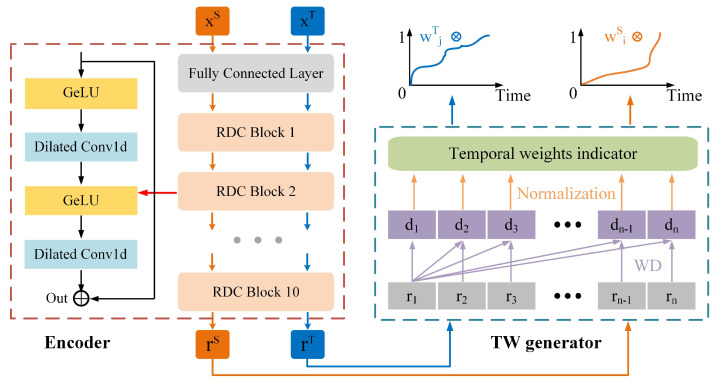
The structure of the Encoder and the mechanism of the temporal weight generator. Orange and blue lines represent two separate paths for source and target domain data. Both are encoded by the Encoder, and then processed by the TW generator to produce temporal weights for each domain.

**Encoder** is designed to transform high-dimensional raw input data from both source and target domains ($x^{S}$ and $x^{T}$) into low-dimensional feature representations ($r^{S}$ and $r^{T}$), improving the efficiency of feature extraction [41]. The proposed Encoder consists of a fully connected layer and 10 sequentially connected *residual dilated convolution* (RDC) blocks, as illustrated in Figure 3. Each RDC block contains two 1-D convolutional layers for feature extraction, two GeLU activation functions to introduce nonlinearity, and a residual connection to mitigate problems such as vanishing or exploding gradients [15,42].

**Predictor** utilizes the low-dimensional feature representations $r^{S}$ and $r^{T}$ learned by the Encoder as input. These representations are processed through three fully connected layers, with the final output: RUL prediction values ${\tilde{y}}^{S}$ and ${\tilde{y}}^{T}$, corresponding to the source and target domains, respectively.

### 3.2. The Temporal Weight Generator

The design of the TW generator is based on the premise that the degradation of a bearing can be reflected by the gradual divergence of feature distributions over time [43]. As the health state deteriorates, the extracted features deviate increasingly from those of the initial healthy state [44]. Therefore, the distribution distance between the feature representation at each time step $r_{i}$ and that at the initial time step $r_{1}$ can serve as an effective indicator of the degradation degree.

Based on this motivation, the TW generator constructs a normalized time indicator $w_{i}$ to capture the health level of a bearing over time. As illustrated in Figure 3, this indicator is derived by measuring the feature distribution distance $d_{i}$, between the feature representation at each time step $r_{i}$ and the feature representation at the initial time step $r_{1}$. These calculated distances $d_{i}$, are then normalized to produce the time indicator $w_{i}$, which ranges from 0 to 1.

In this paper, the WD is used to measure the feature distribution distance $d_{i}$ between $r_{i}$ and $r_{1}$, and its calculation can be described as [31]:

(1) $$\mathrm{WD}=\underset{\gamma ∼\Pi (r_{i},r_{1})}{\inf}E_{(p,q)∼\gamma }\left[∥p-q∥\right]$$

where $\Pi (r_{i},r_{1})$ represents the set of all possible joint distributions between the feature representation at time step $r_{i}$ and the feature representation at the initial time step $r_{1}$. (p,q)∼γ represents a sample from this joint distribution.

After calculating the distance $d_{i}=\mathrm{WD}$ for each time step, min—max normalization is applied to scale these distances into the time indicator $w_{i}$, which lies within the range [0,1] [13]. The formula for this normalization is:

(2) $$w_{i}=\frac{d_{i}-d_{min}}{d_{max}-d_{min}}$$

where $d_{min}$ and $d_{max}$ are the minimum and maximum distances across all time steps.

### 3.3. The Spectral Clustering Module

Time-series data in RUL prediction often exhibit evolving feature distributions over time, as illustrated in Figure 1. Conventional two-stage SDA methods typically divide subdomains with fixed boundaries before performing alignment. However, such predefined and static boundaries fail to adapt to the temporal evolution of feature distributions. Moreover, when subdomains are divided based on disordered features, samples that should belong to the same subdomain may be incorrectly assigned to different ones, resulting in degraded prediction accuracy.

To address these issues, SC is adopted as a graph-based clustering method to identify subdomains in dynamic feature spaces [45]. It first constructs a similarity graph that models pairwise relationships among feature samples and then derives a graph Laplacian to capture the global data structure. By analyzing the spectral properties of this Laplacian, SC embeds samples into a lower-dimensional space, where similar features are more effectively grouped and subdomains are clearly separated.

In RUL prediction, the SC algorithm maximizes similarity within clusters and minimizes similarity across clusters, leading to compact and well-separated subdomains. When integrated with SDA, this clustering strategy reduces feature overlap and preserves the temporal consistency of degradation patterns, thereby enhancing the alignment between source and target domains. Accordingly, the proposed SC module dynamically updates subdomain boundaries during training and periodically reclusters features to improve boundary clarity and subdomain discriminability. The implementation of the proposed SC module involves four steps, which are described as follows:

**Step 1: Initialization of subdomain boundaries.** Although subdomain boundaries can be intuitively described as dividing lines in a two-dimensional space, in implementation they are implicitly represented by a cluster indicator matrix. Since feature representations $r\in R^{N\times V}$ generated by the Encoder capture the degradation patterns of bearings, applying K-means clustering to *r* can group similar features into *K* clusters/subdomains [46,47]. The clustering result is encoded as a one-hot matrix $F\in R^{N\times K}$, where each row $F_{i,:}$ indicates the subdomain assignment of sample *i*. In this way, F implicitly defines the subdomain boundaries: if two samples have similar rows, they are considered to belong to the same subdomain.

**Step 2: Softening of subdomain boundaries.** Subdomain boundaries are initialized using K-means clustering in Step 1, generating a one-hot indicator matrix $F\in R^{N\times K}$. However, the bearing degradation process is continuous and gradual, this hard assignment may lead to misclassification at subdomain boundaries. To address this, soft clustering is more appropriate than one-hot clustering, as it represents the probability that each sample belongs to each subdomain. Therefore, a soft clustering method based on *truncated singular value decomposition* (Truncated SVD) is used to soften the subdomain boundaries [48,49].

Specifically, the feature representations $r\in R^{N\times V}$ are decomposed via Truncated SVD as $r\approx U\Sigma V^{⊤}$, where $U\in R^{N\times K}$, $\Sigma \in R^{K\times K}$, and $V\in R^{V\times K}$. The soft subdomain boundary indicator matrix F is then defined as:

(3) $$F_{i,:}=\frac{{\left(U\Sigma \right)}_{i,:}}{\sum_{k=1}^{K}{\left(U\Sigma \right)}_{i,k}},\;\mathrm{for}\;i=1,\ldots ,N,$$

where $F_{i,:}$ denotes the *i*-th row of F. This row-wise normalization turns each row vector into a probability distribution over *K* subdomains. The matrix UΣ provides a low-dimensional embedding of the samples that capture their principal components.

**Step 3: Clustering of feature representations**. The soft clustering indicator matrix $F\in R^{N\times K}$ obtained in Step 2 not only represents the probability distribution of subdomains, but also serves as a guide matrix to cluster similar features within $r\in R^{N\times V}$. Inspired by spectral clustering, the clustering objective can be reformulated as a trace maximization problem based on the spectral relaxation method, which aims to maximize $\mathrm{Tr}\left(F^{⊤}rr^{⊤}F\right)$. Maximizing this trace encourages F to keep the most important information of the similarity matrix $rr^{⊤}$. As a result, samples from the same subdomain will be projected closer, while samples from different subdomains will be projected further apart. The spectral clustering loss can be defined as:

(4) $$L_{\mathrm{clustering}}=-\mathrm{Tr}\left(F^{⊤}rr^{⊤}F\right)$$

where Tr(·) denotes the matrix trace, $r\in R^{N\times V}$ is feature representations to be clustered, and $F\in R^{N\times K}$ is an orthogonal indicator matrix. Here, *N* is the number of samples, *V* is the number of features in the feature representation, and *K* is the number of clusters.

**Step 4: Iterative update of subdomain boundaries and feature representations**. To dynamically divide subdomain boundaries based on the evolving feature distribution, feature representations *r* and the clustering indicator matrix F are optimized in an iterative manner. In each iteration, when F is fixed, *r* is updated under the guidance of $L_{\mathrm{clustering}}$. This encourages *r* to be more compact within each subdomain and more distinguishable across different subdomains. Conversely, when *r* is fixed, F is updated based on the current feature distribution. This update allows the model to adjust subdomain boundaries that better reflect the evolving similarity relationships among samples. To ensure stable training, F is updated once every 40 epochs, while *r* is updated at every epoch.

### 3.4. The Subdomain Adaptation Module

The SDA module is a critical component of the proposed SC-SAN, designed to bridge the gap between the source and target domains by leveraging *local maximum mean discrepancy* (LMMD). Unlike traditional DA methods that focus on global-scale alignment, the SDA performs alignment at the subdomain level, effectively capturing localized variations in the degradation process across domains.

As illustrated in Figure 2, the Predictor maps the source domain representation $r^{S}$ and target domain representation $r^{T}$ into RUL prediction results, denoted as ${\tilde{y}}^{S}$ and ${\tilde{y}}^{T}$, respectively.

These prediction results are then input into a high-dimensional feature space, where ${\tilde{y}}^{S}$ and ${\tilde{y}}^{T}$ are divided into different subdomains using clustering indicator matrices $F^{S}$ and $F^{T}$ generated by the SC module. Within each subdomain, the feature distribution discrepancies are minimized through optimizing the LMMD loss. During the domain alignment process, TW indicators $w_{i}^{S}$ and $w_{j}^{T}$ are introduced for the source and target domains to emphasize the importance of specific samples based on their temporal relevance within the degradation cycle. By assigning higher weights to critical time steps, the SDA module ensures precise feature alignment while accounting for the temporal sensitivity of the data.

The LMMD is calculated using a Gaussian kernel function ϕ(·), which measures the discrepancies between the distributions of ${\tilde{y}}^{S}$ and ${\tilde{y}}^{T}$ within each subdomain. The LMMD is defined as:

(5) $$\mathrm{LMMD}\left({\tilde{y}}^{S},{\tilde{y}}^{T}\right)=\sum_{k=1}^{K}{∥\frac{1}{N_{k}^{S}}\sum_{i=1}^{N_{k}^{S}}w_{i}^{S}\cdot \phi \left({\tilde{y}}_{i,k}^{S}\right)-\frac{1}{N_{k}^{T}}\sum_{j=1}^{N_{k}^{T}}w_{j}^{T}\cdot \phi \left({\tilde{y}}_{j,k}^{T}\right)∥}_{H}^{2}$$

where *K* represents the number of subdomains/clusters; $w_{i}^{S}$ and $w_{j}^{T}$ denote the temporal weights for source and target domain features, reflecting the temporal importance of individual samples; $N_{k}^{S}$ and $N_{k}^{T}$ denote the number of samples in subdomain *k* for the source and target domains, respectively; H denotes the reproducing kernel Hilbert space; and ϕ(·) is defined as a Gaussian kernel function.

### 3.5. Model Parameters Optimization

Model parameters optimization is guided by three objectives: ① the RUL prediction loss $L_{R}$, which measures the difference between the ground truth and predicted RUL; ② the spectral clustering loss $L_{C}$, which clusters similar features while separating dissimilar features; and ③ the domain distribution loss $L_{D}$, which minimizes the discrepancies between the predicted values in the source and target domains.

**RUL prediction loss**: for the first optimization objective, the *mean square error* (MSE) is used to define the prediction error $L_{R}$, which is commonly used in regression problems [23]. The MSE is expressed as follows:

(6) $$L_{R}=\frac{1}{N^{S}}\sum_{i=1}^{N^{S}}{\left({\tilde{y}}_{i}^{S}-y_{i}^{S}\right)}^{2}$$

where ${\tilde{y}}_{i}^{S}$ and $y_{i}^{S}$ are the predicted RUL values and the ground truth RUL values both from the source domain, respectively. $N^{S}$ is the number of samples in the source domain.

**Spectral clustering loss**: for the second optimization objective, spectral clustering is used to cluster similar feature representations while separating dissimilar ones within both source and target domains. The total clustering loss $L_{C}$ is defined as follows:

(7) $$L_{C}=-\mathrm{Tr}\left({\left(F^{S}\right)}^{⊤}r^{S}{\left(r^{S}\right)}^{⊤}F^{S}\right)-\mathrm{Tr}\left({\left(F^{T}\right)}^{⊤}r^{T}{\left(r^{T}\right)}^{⊤}F^{T}\right)$$

where ${\left(\cdot \right)}^{⊤}$ denotes the matrix transpose; $r^{S}{\left(r^{S}\right)}^{⊤}$ and $r^{T}{\left(r^{T}\right)}^{⊤}$ represent the similarity matrices of the source and target domains; $F^{S}$ and $F^{T}$ are the soft cluster indicator matrices for the source and target domains; and Tr(·) is the trace of a matrix.

**Domain distribution loss**: for the third optimization objective, the LMMD is used to calculate the subdomain loss $L_{D}$, which is expressed as follows:

(8) $$L_{D}=\sum_{k=1}^{K}\mathrm{LMMD}\left(w_{i}^{S}{\left\{{\tilde{y}}_{i}^{S}\right\}}_{i=1}^{N_{k}^{S}},w_{j}^{T}{\left\{{\tilde{y}}_{j}^{T}\right\}}_{j=1}^{N_{k}^{T}}\right)$$

**Total loss**: the total loss L of the proposed SC-SAN integrates the three objectives described above (Equations (6)–(8)) and is formulated as:

(9) $$L=L_{R}+\mu \cdot L_{C}+\lambda \cdot L_{D}$$

where μ, λ denotes the tradeoff parameters, set to 0.1 and 0.1, respectively.

Once the loss function L is defined, the optimal parameters for the proposed SC-SAN can be obtained by optimizing the loss function. The steps involved in this process are summarized in Algorithm 1.
**Algorithm 1** SC-SAN1:**procedure** Main(raw data)2:    **for** $x^{S},x^{T},y^{S},K$ in raw data **do**3:        // Feature extraction:4:        $r^{S},r^{T}⇐$ Encoder $x^{S},x^{T}$;5:        // TW generator:6:        $d_{i}^{S},d_{j}^{T}⇐$ WD calculation $r^{S},r^{T}$;7:        $w_{i}^{S},w_{j}^{T}⇐$ Normalization $d_{i}^{S},d_{j}^{T}$;8:        // RUL prediction:9:        ${\left\{{\tilde{y}}_{i}^{S}\right\}}_{i=1}^{N^{S}},{\left\{{\tilde{y}}_{j}^{T}\right\}}_{j=1}^{N^{T}}⇐$ Predictor $r^{S},r^{T}$;10:        // Calculate prediction loss:11:        $L_{R}⇐$ MSELoss$\left({\left\{{\tilde{y}}_{i}^{S}\right\}}_{i=1}^{N^{S}},{\left\{y_{i}^{S}\right\}}_{i=1}^{N^{S}}\right)$;12:        // Calculate clustering loss:13:        $F^{S},F^{T}⇐$ Initialize cluster indicator matrix *K*;14:        $L_{\mathrm{clustering}}^{S}⇐$ Source clustering loss $F^{S},r^{S}$;15:        $L_{\mathrm{clustering}}^{T}⇐$ Target clustering loss $F^{T},r^{T}$;16:        $L_{C}⇐$ Sum clustering loss $L_{\mathrm{clustering}}^{S},L_{\mathrm{clustering}}^{T}$;17:        **for** *t* in 1 to Max epoch **do**18:           // Update F every 40 epochs:19:           **if** tmod40==0 **then**20:               $F^{S},F^{T}⇐$ Truncated SVD $r^{S},r^{T}$;21:           **end if**22:        **end for**23:        // Calculate domain loss:24:        $L_{D}⇐$ LMMDLoss$\left({\left\{{\tilde{y}}_{i}^{S}\right\}}_{i=1}^{N^{S}},{\left\{{\tilde{y}}_{j}^{T}\right\}}_{j=1}^{N^{T}},w_{i}^{S},w_{j}^{T},K\right)$;25:        L⇐$L_{R}+\mu \cdot L_{C}+\lambda \cdot L_{D}$;26:        // Update model parameters using L27:    **end for**28:**end procedure**

## 4. Case Study

In order to demonstrate the effectiveness of the proposed SC-SAN in accurately predicting RUL, a grid search method is employed to identify the optimal hyperparameter combination for the total loss function L (see Equation (9)) during model updates. Then, ablation and comparison experiments are conducted using the PRONOSTIA dataset [50] to evaluate the RUL prediction accuracy of SC-SAN and related models. Finally, validation experiments based on the XJTU-SY dataset [51] are performed to evaluate the generalization performance of the proposed SC-SAN.

### 4.1. Data Description

The PRONOSTIA dataset (also known as the IEEE PHM 2012 dataset) is a publicly available benchmark specifically designed for studying bearing RUL prediction. It employs multiple sensors to continuously monitor the bearing degradation process under accelerated operating conditions. Vibration signals are captured by two piezoelectric accelerometers mounted on the bearing housing in the horizontal and vertical directions. These sensors convert mechanical vibrations into electrical signals with high sensitivity and a wide frequency bandwidth, enabling the precise detection of early-stage bearing faults. The dataset includes measurements from 17 rolling bearings monitored from healthy operation to complete failure. The vibration data were recorded every 10 s with a sampling frequency of 25.6 kHz. Three different operating conditions are provided, each defined by a specific combination of rotational speed, radial load, and bearing type, as summarized in Table 3.

### 4.2. Model Design

The model training employs the Adam optimizer, with the learning rate adjusted via the StepLR strategy. The initial learning rate is set to 0.001, and a decay factor of 0.1 is applied every 100 epochs. The batch size is set to 1, indicating that the full-life data of a single Bearing is input in each training epoch. The number of subdomains *K* in Equation (5) is empirically set to 3 [25]. The model is trained for a total of 200 epochs based on PyTorch 1.13.0.

Taking Bearing 1_1 from the PRONOSTIA dataset as an example, its full lifespan consists of 2803 time steps (samples), each with 2560 features. First, raw data is fed into the Encoder for representation learning, where the number of features is reduced from 2560 to 320. The learned representations are then passed to the Predictor to estimate the RUL, during which the number of features changes from 320 to 1. Table 4 summarizes the data shape changes at each model layer, along with the parameters of each layer.

### 4.3. Evaluation Metrics for RUL Prediction

The accuracy of RUL prediction is evaluated based on three types of prediction metrics: *mean absolute error* (MAE), *root mean square error* (RMSE) and a Score function commonly used in the RUL prediction tasks [31]. These metrics are defined as follows:

(10) $$\begin{array}{cc} \mathrm{MAE} & =\frac{1}{N}\sum_{i=1}^{N}\left|{\tilde{y}}_{i}-y_{i}\right| \end{array}$$

(11) $$\begin{array}{cc} \mathrm{RMSE} & =\sqrt{\frac{1}{N}\sum_{i=1}^{N}{\left({\tilde{y}}_{i}-y_{i}\right)}^{2}} \end{array}$$

(12) $$\begin{array}{cc} \mathrm{Score} & =\frac{1}{N-1}\sum_{i=1}^{N-1}A_{i} \end{array}$$

where:

(13) $$\begin{array}{cc} A_{i} & =\left\{\begin{array}{cc} e^{-\ln\left(0.5\right)\cdot \left(Er_{i}/5\right)} & \mathrm{if}\;Er_{i}\leq 0\\ e^{+\ln\left(0.5\right)\cdot \left(Er_{i}/20\right)} & \mathrm{if}\;Er_{i}>0 \end{array}\right. \end{array}$$

(14) $$\begin{array}{cc} Er_{i} & =\frac{y_{i}-{\tilde{y}}_{i}}{y_{i}}\times 100 \end{array}$$

where $y_{i}$ and ${\tilde{y}}_{i}$ represent the ground truth RUL and predicted RUL respectively, *N* is the number of samples in the testing data.

### 4.4. Discussion of Hyperparameters Settings

**Figure 4:**
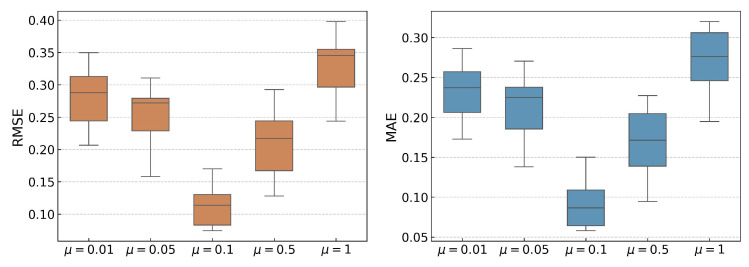
RMSE and MAE results for varying μ values with λ fixed at 0.1.

**Figure 5:**
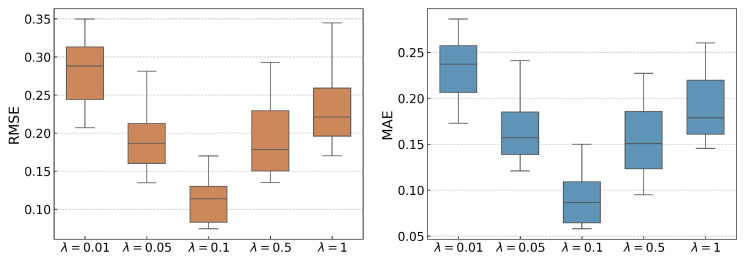
RMSE and MAE results for varying λ values with μ fixed at 0.1.

The influence of the hyperparameters μ and λ on RUL prediction is examined. A grid search over [0.01,0.05,0.1,0.5,1] indicates that μ=0.1 and λ=0.1 achieve the best performance. To verify that this combination is not just a local optimum, each parameter is varied individually while keeping the other fixed, with 10 repetitions to reduce randomness (see Figure 4 and Figure 5).

When λ is fixed at 0.1, increasing μ from 0.01 to 0.1 leads to a noticeable reduction in prediction errors. This improvement is due to an increased focus on clustering similar features, as more weight is given to the spectral clustering loss $L_{C}$. This encourages SC-SAN to conduct downstream tasks based on well-clustered feature distributions. However, as μ increases further from 0.1 to 1, the prediction accuracy declines. This is because SC-SAN allocates excessive learning capacity to optimizing $L_{C}$, which diminishes its focus on the core prediction loss $L_{R}$. As a result, while the feature representations may align better with clustering objectives, the overall prediction accuracy decreases due to the shift in focus from prediction to clustering.

Similarly, Figure 5 illustrates the trends of RMSE and MAE with varying values of λ. When μ is fixed at 0.1, increasing λ from 0.01 to 0.1 results in a reduction in prediction error. This suggests that increasing the weight of the domain distribution loss $L_{D}$ in the total loss function Equation (9) helps SC-SAN focus on minimizing the feature distribution discrepancies between the source and target domains. However, when λ exceeds 0.1, the prediction errors rise again. This indicates that overemphasizing $L_{D}$ can lead SC-SAN to prioritize domain transfer at the cost of learning the essential mapping between features and labels, which is essential for accurate RUL prediction.

### 4.5. Ablation Experiments of the Proposed Model

The proposed SC-SAN comprises three auxiliary modules: ① a TW generator, ② an SC module, and ③ an SDA module. To evaluate the contribution of each module, ablation experiments are performed across six transfer tasks using the PRONOSTIA dataset. Details of the training and testing data for these tasks are summarized in Table 5. The model configurations for the ablation experiments are defined as follows:

- w/o SDA: Model A excludes the SDA module and relies on traditional DA, aligning feature distributions on a global scale.
- w/o TW: Model B excludes the TW generator, assigning equal weights to all features within each subdomain during domain alignment.
- w/o SC: Model C excludes the SC module, meaning the model does not update parameters with the goal of clustering similar features.
- Model D represents the proposed SC-SAN.

**Figure 6:**
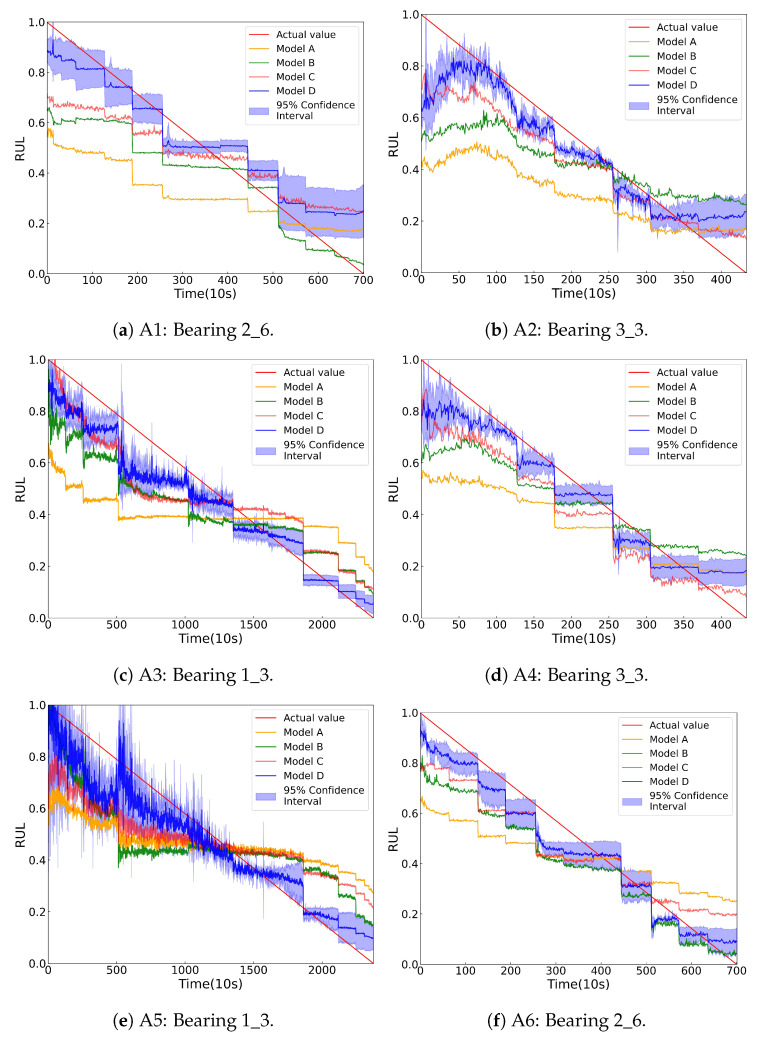
Prediction results for the six transfer tasks in Table 5.

To mitigate the impact of randomness, all experiments are repeated 10 times. Table 6 presents the RUL prediction metrics for the four models across the six transfer tasks. Figure 6 shows the RUL prediction results for each model across the tasks.

As shown in Table 6, Model A exhibits the highest prediction errors in terms of MAE and RMSE, and the lowest Score, highlighting the critical role of the SDA module in improving prediction accuracy. By dividing the data into distinct subdomains, the SDA module enables the model to focus on the unique features of each subdomain, thereby achieving precise domain alignment and improving prediction performance. Model B achieves a higher prediction Score than Model A, underscoring the importance of assigning different weights to samples through a TW indicator in RUL prediction. Without the TW generator, Model B fails to emphasize key degradation phase features during domain alignment, resulting in insufficient learning of degradation patterns, which are essential for accurate RUL predictions. Model C outperforms Model A and Model B in the prediction Score but is inferior to Model D, indicating that the SC module contributes to enhanced RUL prediction. However, its impact on accuracy is relatively modest compared to the TW generator and the SDA module. Although the SC module helps group similar features together, its introduction could shift the model’s attention towards feature similarity rather than effectively learning degradation patterns or performing domain alignment.

**Table 5:** The six transfer tasks based on the PRONOSTIA dataset.

Task	Conditions	Training Bearings	Test Bearings
A1	C1→C2	Labeled: Bearing 1_1	Bearing 2_6
		Unlabeled: Bearing 2_1	
A2	C1→C3	Labeled: Bearing 1_1	Bearing 3_3
		Unlabeled: Bearing 3_1	
A3	C2→C1	Labeled: Bearing 2_1	Bearing 1_3
		Unlabeled: Bearing 1_1	
A4	C2→C3	Labeled: Bearing 2_1	Bearing 3_3
		Unlabeled: Bearing 3_1	
A5	C3→C1	Labeled: Bearing 3_1	Bearing 1_3
		Unlabeled: Bearing 1_1	
A6	C3→C2	Labeled: Bearing 3_1	Bearing 2_6
		Unlabeled: Bearing 2_1	

**Table 6:** The RUL prediction metrics of Models A–D for the six transfer tasks in Table 5.

Tasks	Metrics	Model A	Model B	Model C	Model D
		w/o SDA	w/o TW	w/o SC	Proposed Model
A1	MAE	0.2465 ± 0.0318	0.1479 ± 0.0594	0.1224 ± 0.0367	**0.0782 ± 0.0209**
	RMSE	0.3277 ± 0.0412	0.1924 ± 0.0499	0.1595 ± 0.0436	**0.0961 ± 0.0332**
	Score	0.2157 ± 0.0346	0.3121 ± 0.0563	0.4356 ± 0.0581	**0.4979 ± 0.0652**
A2	MAE	0.2325 ± 0.0267	0.1892 ± 0.0498	0.1363 ± 0.0442	**0.0913 ± 0.0271**
	RMSE	0.2816 ± 0.0325	0.2192 ± 0.0427	0.1603 ± 0.0432	**0.1244 ± 0.0273**
	Score	0.2071 ± 0.0296	0.2768 ± 0.0532	0.3721 ± 0.0596	**0.4796 ± 0.0461**
A3	MAE	0.2053 ± 0.0687	0.1271 ± 0.0299	0.1365 ± 0.0395	**0.0822 ± 0.0267**
	RMSE	0.2526 ± 0.0711	0.1653 ± 0.0336	0.1574 ± 0.0691	**0.1012 ± 0.0413**
	Score	0.2667 ± 0.0690	0.4038 ± 0.0519	0.3444 ± 0.0753	**0.5027 ± 0.0687**
A4	MAE	0.2121 ± 0.0264	0.1540 ± 0.0713	0.1030 ± 0.0216	**0.0711 ± 0.0185**
	RMSE	0.2525 ± 0.0379	0.1791 ± 0.0612	0.1310 ± 0.0345	**0.0849 ± 0.0266**
	Score	0.2745 ± 0.0311	0.3611 ± 0.0689	0.4519 ± 0.0471	**0.5494 ± 0.0314**
A5	MAE	0.2227 ± 0.0486	0.1672 ± 0.0537	0.1470 ± 0.0213	**0.0796 ± 0.0298**
	RMSE	0.2948 ± 0.0546	0.2041 ± 0.0533	0.1633 ± 0.0382	**0.1014 ± 0.0274**
	Score	0.2460 ± 0.0479	0.3156 ± 0.0829	0.3024 ± 0.0389	**0.4845 ± 0.0328**
A6	MAE	0.1944 ± 0.0472	0.1214 ± 0.0404	0.1174 ± 0.0264	**0.0733 ± 0.0126**
	RMSE	0.2475 ± 0.0491	0.1509 ± 0.0386	0.1360 ± 0.0345	**0.0891 ± 0.0234**
	Score	0.2610 ± 0.2120	0.4333 ± 0.0714	0.4347 ± 0.0648	**0.5138 ± 0.0294**

In task A5, although Model C exhibits lower RMSE and MAE than Model B, its score is lower. This discrepancy arises because the Score function in Equation (12) is more tolerant to negative errors (underestimation) and more penalizing to positive errors (overestimation). Therefore, if most errors in this task are negative, Model B will obtain a higher score despite having a higher prediction error. In task A3, Model B has a lower MAE than Model C, but a higher RMSE. Furthermore, these prediction curves all exhibit discontinuities. This is because bearing degradation typically occurs in multiple stages rather than following a smooth, continuous trend. In real industrial environments, external shocks, load changes, or environmental disturbances often cause sudden accelerations in the degradation process. These sudden degradations result in jumps in the health state rather than gradual ones. The Encoder successfully captures these sudden degradation patterns during training, and these jumps are reflected in the predicted RUL curves.

### 4.6. Comparison with Related RUL Prediction Models

**Figure 7:**
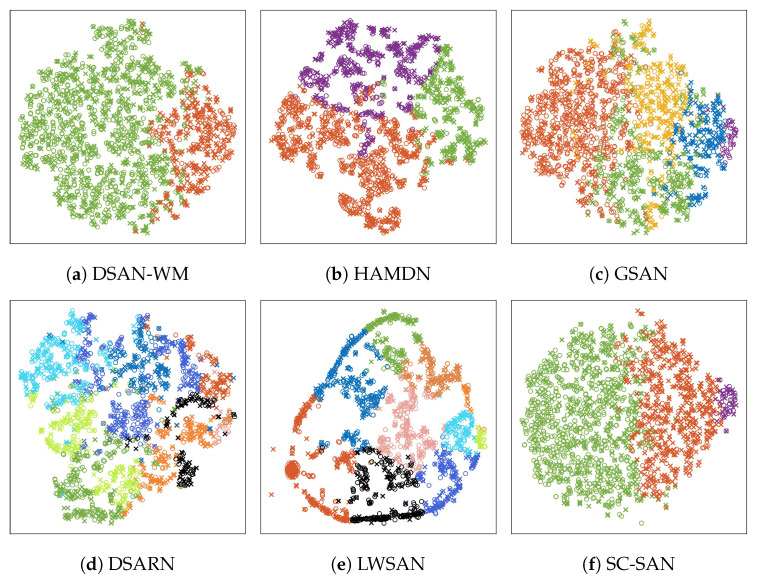
T-SNE visualization results of all SDA models listed in Table 7. ‘O’ and ‘X’ represent the source and target domains. Different colors indicate different subdomains, where (**a**) shows 2 subdomains, (**b**) shows 3 subdomains, (**c**) shows 5 subdomains, (**d**,**e**) show 10 subdomains, and (**f**) shows 3 subdomains.

The performance of the proposed SC-SAN is evaluated through four categories of existing models: ① metric-based DA models, such as TCNN [20] and TLMAN [21]; ② adversarial-based DA models, including MADA [22] and MCDA [23]; ③ two-stage SDA models, including DSAN-WM [26], HAMDN [25], and GSAN [24]; and ④ end-to-end SDA models, such as DSARN [13] and LWSAN [28]. These models are tested on six transfer scenarios listed in Table 5, and the average performance in terms of MAE, RMSE, and Score is summarized in Table 7. To further illustrate the effectiveness of subdomain alignment, T-SNE visualizations of the feature distributions for all SDA models are presented in Figure 7.

The results in Table 7 reveal that two metric-based DA models exhibit the weakest prediction performance. This can be attributed to their reliance on MMD or MK-MMD for global domain alignment without adding additional regularization terms or employing refined subdomain alignment strategies. In contrast, the two adversarial-based DA models outperform the metric-based models, indicating that introducing regularization terms is an effective strategy to handle domain shifts. Specifically, MADA improves RUL prediction accuracy by employing a contrastive learning framework with a positive pair matching module as a regularization term in the loss function, which is conceptually similar to the SC module proposed in this paper. MCDA enhances predictive performance by introducing a regularization term into its adversarial network, enabling the alignment of conditional and marginal distributions.

Among the SDA models, two-stage models perform worse than end-to-end models, mainly because the subdomain division errors introduced in the first stage are propagated and accumulated in the alignment phase. According to Figure 7a–c, as the number of subdomains increases, the clustering boundaries become increasingly blurred. By contrast, although end-to-end models may introduce misclassification errors when generating pseudo-labels, these errors can potentially be corrected through a jointly optimized loss function. The proposed SC-SAN achieves the best overall performance. Its superiority stems from the SC module that ensures dynamic subdomain division and the effective weighting of key samples by the TW generator. Comparing Figure 7d,e with Figure 7f, it can be seen that dynamically dividing subdomains based on evolving feature distributions can obtain more discriminative subdomain boundaries. As shown in Table 7, the model achieves an average inference latency of only 15.32 milliseconds per sample on an Intel Core i5-12500H processor, well below the real-time processing threshold of 50 milliseconds, thereby meeting the deployment requirements of many industrial applications.

### 4.7. Validation of Model Generalization Performance

To validate the generalization performance of the proposed SC-SAN, the XJTU-SY dataset is also utilized. In the XJTU-SY test rig, vibration signals are acquired using two miniature accelerometers (PCB 352C33, PCB Piezotronics, Depew, NY, USA) mounted orthogonally on the bearing housing, with one sensor in the horizontal direction and the other in the vertical direction. These accelerometers convert mechanical vibrations into electrical signals with high sensitivity and a wide frequency response, enabling precise detection of early-stage bearing faults. The vibration signals are sampled at 25.6 kHz, with each session recording 32,768 data points (1.28 s of data) at regular intervals throughout the bearings’ full lifetimes. The dataset contains measurements from 15 rolling bearings monitored from healthy operation to failure, with each bearing operating under a unique combination of rotational speed and radial load, as detailed in Table 8.

The proposed SC-SAN is also compared with four different types of models in Table 7: ① metric-based DA models, including TCNN [20] and TLMAN [21]; ② adversarial-based DA models, including MADA [22] and MCDA [23]; ③ two-stage SDA models, including DSAN-WM [26], HAMDN [25] and GSAN [24]; and ④ end-to-end SDA models, including DSARN [13] and LWSAN [28].

**Figure 8:**
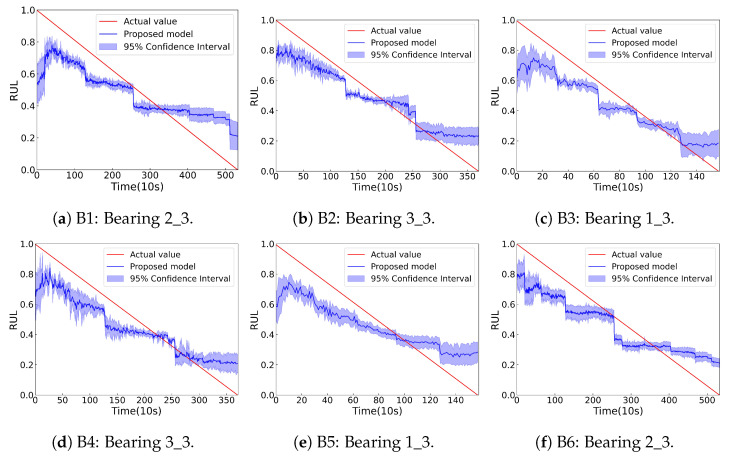
Prediction results of the proposed SC-SAN on the six transfer tasks in Table 9.

All these models are evaluated across six transfer scenarios based on the XJTU-SY dataset, as summarized in Table 9. To mitigate the impact of randomness, all experiments are repeated 10 times. The average RUL prediction metrics of the these models are presented in Table 10. Furthermore, Figure 8 displays the RUL prediction results of the proposed SC-SAN.

As shown in Figure 8, the predictive results of the proposed SC-SAN are close to the ground truth, confirming its robust generalization performance. These predicted curves exhibit varying degrees of fluctuation. This fluctuation is consistent with the actual physical degradation behavior of the bearing and demonstrates the ability of SC-SAN to learn and represent true degradation patterns. Furthermore, the average results in Table 10 show that SC-SAN achieves the highest Scores and the lowest prediction errors compared to other models, indicating that the integration of the time-weight generation module and the clustering-aware module significantly enhances RUL prediction accuracy. As shown in Table 10, the model achieves an average inference latency of only 20.8 milliseconds per sample on an Intel Core i5-12500H processor, meeting the deployment requirements of many industrial applications.

## 5. Discussion

### 5.1. The Choice of Temporal Weight Construction Methods

In this work, the temporal weight generator constructs a normalized time indicator $w_{i}$ by measuring the distributional distance between the feature representation at each time step $r_{i}$ and the initial healthy state $r_{1}$. Three most commonly used distance metrics were considered for constructing $w_{i}$:

- WD: captures the geometric shift in feature distributions over time.
- MMD: measures the difference between distributions in a reproducing kernel Hilbert space [25].
- *Pearson correlation coefficient* (PCC): quantifies linear correlation with the reference state [21].

**Figure 9:**
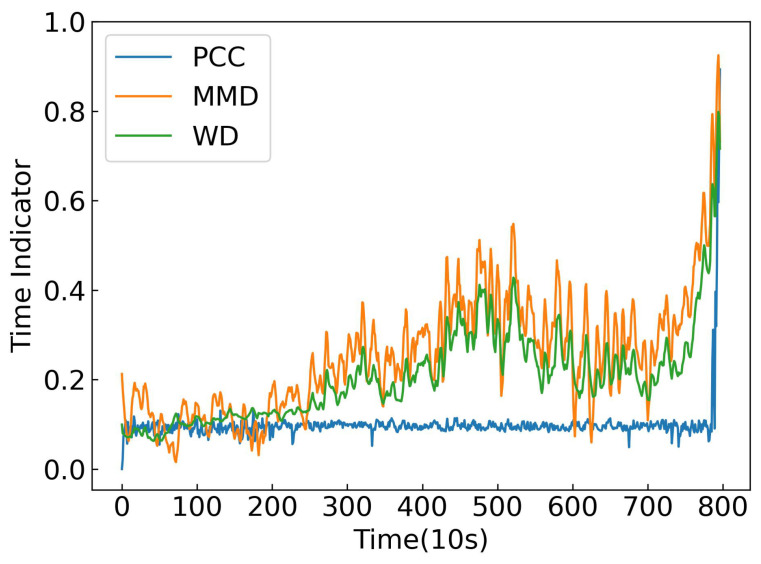
Comparison of time indicator construction methods.

The effectiveness of these metrics was evaluated using Bearing 2_2 from the PRONOSTIA dataset and time indicators are shown in Figure 9. The results indicate that WD produces the most monotonic and smooth progression of the time indicator, closely following the actual degradation trend. In contrast, MMD and PCC either overreact to noise or fail to fully capture the distributional evolution. These findings demonstrate that WD is the most suitable choice for constructing temporal weights and modeling the health state of bearings in this work.

To further assess the sensitivity of WD estimation on small samples, three quantitative metrics were employed. Monotonicity evaluates how steadily the health indicator decreases over time [52], Correlation measures its alignment with the actual degradation trend [53], and Robustness reflects resistance to random fluctuations [54]. As shown in Table 11, WD attains the highest values across all metrics, demonstrating a smooth and monotonic progression, strong trend alignment, and robust behavior. These findings confirm that WD is highly suitable for constructing time weights in high-dimensional, noisy scenarios, maintaining stability even with limited sample sizes.

### 5.2. The Choice of Dilated CNN

To justify the choice of dilated CNN as the feature extractor in SC-SAN, a comparative study is conducted with two popular sequence modeling architectures: *long short-term memory* (LSTM) and Transformer.

- LSTM: captures long-term dependencies in sequences but processes data sequentially, leading to lower efficiency and potential gradient vanishing in long sequences.
- Transformer: models global dependencies via self-attention but has quadratic computational complexity, which can be costly for long industrial time series.
- Dilated CNN: used in SC-SAN, efficiently captures both short- and long-term dependencies with exponentially expanding receptive fields, allows parallel computation, and maintains linear complexity.

**Figure 10:**
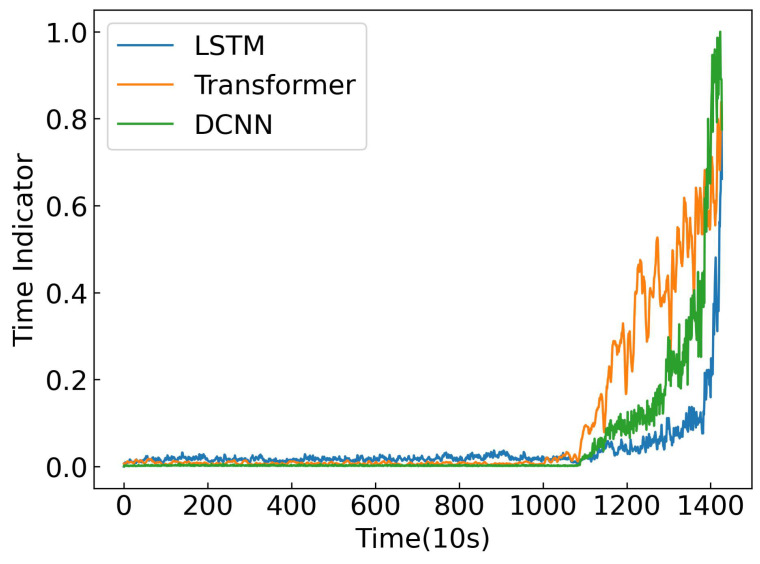
Comparison of feature extraction methods.

Bearing 1_4 from the PRONOSTIA dataset was used to construct the time indicator, and the resulting indicators are shown in Figure 10. Performance was evaluated using Monotonicity, Correlation, and Robustness metrics. As summarized in Table 11, the dilated CNN consistently outperforms both LSTM and Transformer across all three metrics. These results demonstrate that the dilated CNN achieves an effective balance between capturing long-range temporal dependencies and maintaining computational efficiency, making it particularly well-suited for industrial RUL prediction tasks.

### 5.3. Sensitivity Analysis of K Values

**Figure 11:**
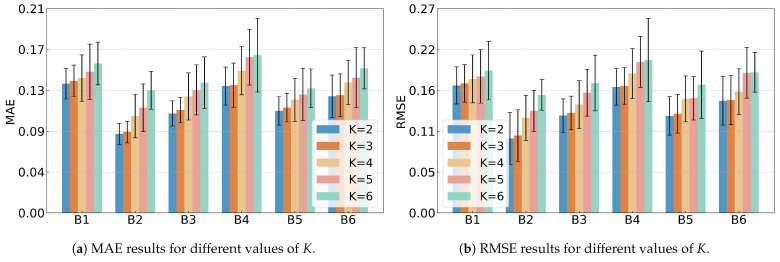
MAE and RMSE results for varying *K* values.

A sensitivity analysis on the value of *K* was conducted based on the XJTU-SY dataset, and the results are shown in Table 12 and Figure 11. The performance metrics (MAE, RMSE, and Score) remain relatively stable across different *K* values, showing that SC-SAN is not sensitive to *K*. Smaller *K* values tend to give slightly better predictions, as fewer clusters capture the overall trend more accurately.

The value K=3 was selected based on the common understanding of bearing or machinery degradation, which is usually divided into three stages: healthy, mildly degraded, and severely degraded. Setting K=3 aligns with the semantic meaning of this study and makes the model outputs more interpretable.

## 6. Conclusions

This paper proposes a SC-SAN, which integrates a backbone RUL prediction network, a TW generator, an SC module, and an SDA module. Experimental results based on two bearing datasets, including ablation, comparison, and validation experiments, highlight the following key findings: ① SC-SAN adaptively adjusts subdomain division boundaries, achieving fine-grained subdomain alignment. ② SC-SAN effectively clusters similar features while separating dissimilar features, ensuring precise subdomain division. ③ By assigning greater importance to degradation-related features during SDA, SC-SAN outperforms state-of-the-art models.

Despite its strengths, SC-SAN faces challenges in real industrial deployment. Degradation in real equipment occurs over long periods, and components cannot be allowed to fail, so only partial degradation segments are available for training. Combined with variability in operating conditions and measurement noise, these factors may limit subdomain alignment and RUL prediction accuracy. Future work will focus on adapting SC-SAN to handle partial, heterogeneous, and noisy industrial data, ensuring robust performance across diverse scenarios.

## Figures and Tables

**Table 1:** Comparison of the proposed model with related models.

Method	Alignment Type	Adaptation Method	Model Type
TCNN [20]	DA	Metric-based	End-to-end model
TLMAN [21]	DA	Metric-based	Two-stage model
MADA [22]	DA	Adversarial-based	End-to-end model
MCDA [23]	DA	Adversarial-based	End-to-end model
GSAN [24]	SDA	Manifold distribution	Two-stage model
HAMDN [25]	SDA	Similarity metric	Two-stage model
DSAN-WM [26]	SDA	Classification network	Two-stage model
DSARN [27]	SDA	Discretized continuous label	End-to-end model
LWSAN [28]	SDA	Discretized continuous label	End-to-end model
Proposed SC-SAN	SDA	Spectral clustering	End-to-end model

**Table 2:** List of frequently used symbols and their definitions.

Symbol	Definition
*x*	Full time-series data representing the entire lifespan of a bearing
*r*	Feature representations generated by the Encoder
*N*	Number of time lengths (samples) in the bearing data
*M*	Number of features in raw data
*V*	Number of features in a feature representation (V<M)
*K*	Number of clusters
$d_{i}$	Feature distribution distance between the feature representation at time step $r_{i}$ and $r_{1}$
$w_{i}$	Normalized time weights reflecting bearing health over time
F	Cluster indicator matrix
${\tilde{y}}^{S},y^{S}$	Source domain RUL prediction values and ground truth values, respectively

**Table 3:** Description of the PRONOSTIA dataset.

	Condition 1	Condition 2	Condition 3
Load (N)	4000	4200	5000
Speed (rpm)	1800	1650	1500
Bearings	Bearing 1_1 ∼ 1_7	Bearing 2_1 ∼ 2_7	Bearing 3_1 ∼ 3_3

**Table 4:** The architecture parameters of the proposed SC-SAN.

Type	Shape	In/Out Features	Kernel	Dilation	Padding
Linear	(2803,2560)→(2803,256)	2560/256	−	−	−
Unsqueeze	(2803,256)→(1,2803,256)	−	−	−	−
Transpose	(1,2803,256)→(1,256,2803)	−	−	−	−
RDC Block 1	(1,256,2803)→(1,256,2803)	256/256	3	1	1
RDC Block 2	(1,256,2803)→(1,256,2803)	256/256	3	2	2
RDC Block 3	(1,256,2803)→(1,256,2803)	256/256	3	4	4
RDC Block 4	(1,256,2803)→(1,256,2803)	256/256	3	8	8
RDC Block 5	(1,256,2803)→(1,256,2803)	256/256	3	16	16
RDC Block 6	(1,256,2803)→(1,256,2803)	256/256	3	32	32
RDC Block 7	(1,256,2803)→(1,256,2803)	256/256	3	64	64
RDC Block 8	(1,256,2803)→(1,256,2803)	256/256	3	128	128
RDC Block 9	(1,256,2803)→(1,256,2803)	256/256	3	256	256
RDC Block 10	(1,256,2803)→(1,320,2803)	256/320	3	512	512
Transpose	(1,320,2803)→(1,2803,320)	−	−	−	−
Squeeze	(1,2803,320)→(2803,320)	−	−	−	−
Linear	(2803,320)→(2803,128)	320/128	−	−	−
Linear	(2803,128)→(2803,32)	128/32	−	−	−
Linear	(2803,32)→(2803,1)	32/1	−	−	−

**Table 7:** The average results of comparison with related models based on six transfer scenarios in Table 5, including inference latency per sample.

Type	Model	MAE	RMSE	Score	Inference Time
Metric-based DA	TCNN [20]	0.1962 ± 0.0415	0.2345 ± 0.0532	0.2874 ± 0.0679	17.8 ms/sample
	TLMAN [21]	0.1877 ± 0.0369	0.2162 ± 0.0484	0.3033 ± 0.0617	18.2 ms/sample
Adversarial-based DA	MCDA [23]	0.1050 ± 0.0298	0.1353 ± 0.0412	0.4462 ± 0.0521	22.1 ms/sample
	MADA [22]	0.0796 ± 0.0255	0.0998 ± 0.0329	0.5014 ± 0.0487	20.3 ms/sample
Two-stage SDA	DSAN-WM [26]	0.1487 ± 0.0333	0.1886 ± 0.0448	0.3253 ± 0.0596	20.2 ms/sample
	HAMDN [25]	0.1087 ± 0.0284	0.1369 ± 0.0389	0.4417 ± 0.0520	18.6 ms/sample
	GSAN [24]	0.0937 ± 0.0263	0.1154 ± 0.0342	0.4739 ± 0.0498	19.7 ms/sample
End-to-end SDA	DSARN [13]	0.1137 ± 0.0302	0.1411 ± 0.0395	0.3747 ± 0.0576	25.4 ms/sample
	LWSAN [28]	0.0813 ± 0.0271	0.1065 ± 0.0351	0.4923 ± 0.0475	22.6 ms/sample
Proposed	SC-SAN	**0.0793 ± 0.0226**	**0.0995 ± 0.0299**	**0.5047 ± 0.0456**	**15.3 ms/sample**

**Table 8:** Description of the XJTU-SY bearing dataset.

	Condition 1	Condition 2	Condition 3
Load (kN)	12	11	10
Speed (rpm)	2100	2250	2400
Bearings	Bearing 1_1 ∼ 1_5	Bearing 2_1 ∼ 2_5	Bearing 3_1 ∼ 3_5

**Table 9:** The six transfer tasks of the XJTU-SY dataset.

Task	Conditions	Training Bearings	Test Bearings
B1	C1→C2	Labeled: Bearing 1_1	Bearing 2_3
		Unlabeled: Bearing 2_1	
B2	C1→C3	Labeled: Bearing 1_1	Bearing 3_3
		Unlabeled: Bearing 3_1	
B3	C2→C1	Labeled: Bearing 2_1	Bearing 1_3
		Unlabeled: Bearing 1_1	
B4	C2→C3	Labeled: Bearing 2_1	Bearing 3_3
		Unlabeled: Bearing 3_1	
B5	C3→C1	Labeled: Bearing 3_1	Bearing 1_3
		Unlabeled: Bearing 1_1	
B6	C3→C2	Labeled: Bearing 3_1	Bearing 2_3
		Unlabeled: Bearing 2_1	

**Table 10:** Validation results in comparison with related methods averaged over six transfer tasks in Table 9, including inference latency per sample.

Type	Model	MAE	RMSE	Score	Inference Time
Metric-based DA	TCNN [20]	0.1950 ± 0.0474	0.2427 ± 0.0459	0.3005 ± 0.0538	21.4 ms/sample
	TLMAN [21]	0.1868 ± 0.0348	0.2203 ± 0.0432	0.3088 ± 0.0517	23.3 ms/sample
Adversarial-based DA	MCDA [23]	0.1458 ± 0.0381	0.1755 ± 0.0476	0.3769 ± 0.0469	26.5 ms/sample
	MADA [22]	0.1296 ± 0.0294	0.1598 ± 0.0332	0.3914 ± 0.0437	25.9 ms/sample
Two-stage SDA	DSAN-WM [26]	0.1513 ± 0.0323	0.1963 ± 0.0478	0.3701 ± 0.0499	25.7 ms/sample
	HAMDN [25]	0.1506 ± 0.0376	0.1879 ± 0.0459	0.3751 ± 0.0578	23.1 ms/sample
	GSAN [24]	0.1391 ± 0.0262	0.1688 ± 0.0327	0.3821 ± 0.0452	24.8 ms/sample
End-to-end SDA	DSARN [13]	0.1761 ± 0.0314	0.2343 ± 0.0421	0.3299 ± 0.0526	29.2 ms/sample
	LWSAN [28]	0.1363 ± 0.0267	0.1600 ± 0.0335	0.3846 ± 0.0443	27.5 ms/sample
Proposed	SC-SAN	**0.1224 ± 0.0261**	**0.1466 ± 0.0344**	**0.4055 ± 0.0468**	**20.8 ms/sample**

**Table 11:** Comparison of time indicator construction methods and feature extraction methods using Monotonicity, Correlation, and Robustness.

Method	Mon	Corr	Rob
PCC	0.21	0.82	0.72
MMD	0.32	0.88	0.63
WD	0.37	0.91	0.74
Transformer	0.42	0.75	0.76
LSTM	0.47	0.80	0.81
DCNN	0.52	0.83	0.87

**Table 12:** Performance of SC-SAN under different values of K on the six transfer tasks in Table 9.

	Metrics	B1	B2	B3	B4	B5	B6
K = 2	MAE	**0.1351 ± 0.0160**	**0.0825 ± 0.0112**	**0.1039 ± 0.0131**	**0.1326 ± 0.0199**	**0.1067 ± 0.0149**	**0.1219 ± 0.0223**
	RMSE	**0.1683 ± 0.0245**	**0.0983 ± 0.0342**	**0.1286 ± 0.0220**	**0.1663 ± 0.0241**	**0.1281 ± 0.0255**	**0.1479 ± 0.0320**
	Score	**0.3939 ± 0.0373**	**0.4925 ± 0.0300**	**0.4519 ± 0.0328**	**0.3953 ± 0.0437**	**0.4384 ± 0.0348**	**0.4029 ± 0.0466**
K = 3	MAE	0.1380 ± 0.0165	0.0845 ± 0.0113	0.1075 ± 0.0130	0.1335 ± 0.0231	0.1101 ± 0.0150	0.1230 ± 0.0223
	RMSE	0.1710 ± 0.0247	0.1021 ± 0.0340	0.1320 ± 0.0222	0.1675 ± 0.0240	0.1310 ± 0.0255	0.1490 ± 0.0325
	Score	0.3980 ± 0.0375	0.4907 ± 0.0300	0.4550 ± 0.0325	0.3960 ± 0.0438	0.4390 ± 0.0348	0.4035 ± 0.0465
K = 4	MAE	0.1410 ± 0.0243	0.1012 ± 0.0228	0.1217 ± 0.0246	0.1485 ± 0.0252	0.1182 ± 0.0225	0.1363 ± 0.0231
	RMSE	0.1769 ± 0.0317	0.1256 ± 0.0299	0.1432 ± 0.0315	0.1839 ± 0.0330	0.1506 ± 0.0303	0.1600 ± 0.0301
	Score	0.3792 ± 0.0594	0.4208 ± 0.0681	0.4026 ± 0.0743	0.3767 ± 0.0379	0.4067 ± 0.0624	0.3821 ± 0.0367
K = 5	MAE	0.1475 ± 0.0289	0.1099 ± 0.0249	0.1286 ± 0.0262	0.1627 ± 0.0291	0.1237 ± 0.0274	0.1413 ± 0.0315
	RMSE	0.1801 ± 0.0352	0.1346 ± 0.0273	0.1587 ± 0.0309	0.1993 ± 0.0337	0.1514 ± 0.0288	0.1850 ± 0.0336
	Score	0.3594 ± 0.0814	0.4192 ± 0.0485	0.3905 ± 0.0625	0.3552 ± 0.0457	0.4005 ± 0.0473	0.3675 ± 0.0698
K = 6	MAE	0.1562 ± 0.0223	0.1281 ± 0.0197	0.1362 ± 0.0270	0.1649 ± 0.0385	0.1302 ± 0.0202	0.1511 ± 0.0215
	RMSE	0.1879 ± 0.0387	0.1558 ± 0.0203	0.1714 ± 0.0368	0.2018 ± 0.0551	0.1692 ± 0.0445	0.1857 ± 0.0263
	Score	0.3712 ± 0.0415	0.3970 ± 0.0335	0.3804 ± 0.0586	0.3668 ± 0.0528	0.3896 ± 0.0553	0.3705 ± 0.0305

## Data Availability

The data presented in this study are openly available through GitHub at https://github.com/WangBiaoXJTU/xjtu-sy-bearing-datasets (accessed on 10 October 2025), reference number [51]; and through the NASA Prognostics Data Repository at https://phm-datasets.s3.amazonaws.com/NASA/10.+FEMTO+Bearing.zip (accessed on 10 October 2025), reference number [50]. No new data were created or analyzed in this study.

## Abbreviations

The following abbreviations are used in this manuscript:

DA	domain adaptation
DANN	domain adversarial neural network
DSAN-WM	deep subdomain adaptation network with weighted multi-source domain
DSARN	deep subdomain adaptive regression network
GSAN	Graph-embedded subdomain adaptation network
HAMDN	hierarchical adaptive multistage degradation network
LMMD	local maximum mean discrepancy
LSTM	long short-term memory
LWSAN	local weighted deep sub-domain adaptation network
MADA	metric adversarial domain adaptation
MAE	mean absolute error
MCDA	multi-constrained domain adaptation
MK-MMD	multi-kernel maximum mean discrepancy
MMD	maximum mean discrepancy
MSE	mean square error
PCC	pearson correlation coefficient
RDC	residual dilated convolution
RMSE	root mean square error
RUL	remaining useful life
SC	spectral clustering
SC-SAN	subdomain adaptation network driven by spectral clustering
SDA	subdomain adaptation
TCNN	transferable convolutional neural network
TLMAN	transferable LSTM multi-channel attention network
Truncated SVD	truncated singular value decomposition
TW	temporal weight
WD	Wasserstein distance
